# The Insulin Receptor: A Potential Target of Amarogentin Isolated from *Gentiana rigescens* Franch That Induces Neurogenesis in PC12 Cells

**DOI:** 10.3390/biomedicines9050581

**Published:** 2021-05-20

**Authors:** Lihong Cheng, Hiroyuki Osada, Tianyan Xing, Minoru Yoshida, Lan Xiang, Jianhua Qi

**Affiliations:** 1 College of Pharmaceutical Sciences, Zhejiang University, Hangzhou 310058, China; clh83787711@zju.edu.cn; 2 Chemical Biology Research Group, RIKEN Center for Sustainable Resource Science, Wako-shi, Saitama 351-0198, Japan; hisyo@riken.jp; 3 School of Pharmacy, Hubei University of Chinese Medicine, Wuhan 430065, China; tongsherry@sina.cn; 4 Chemical Genomics Research Group, RIKEN Center for Sustainable Resource Science, Wako-shi, Saitama 351-0198, Japan; yoshidam@riken.jp; 5 Department of Biotechnology and Collaborative Research Institute for Innovative Microbiology, The University of Tokyo, Bunkyo-ku, Tokyo 113-0033, Japan

**Keywords:** neurodegenerative disease, aging, Alzheimer’s disease, insulin receptor, target identification

## Abstract

Amarogentin (AMA) is a secoiridoid glycoside isolated from the traditional Chinese medicine, *Gentiana rigescens* Franch. AMA exhibits nerve growth factor (NGF)-mimicking and NGF-enhancing activities in PC12 cells and in primary cortical neuron cells. In this study, a possible mechanism was found showing the remarkable induction of phosphorylation of the insulin receptor (INSR) and protein kinase B (AKT). The potential target of AMA was predicted by using a small-interfering RNA (siRNA) and the cellular thermal shift assay (CETSA). The AMA-induced neurite outgrowth was reduced by the siRNA against the INSR and the results of the CETSA suggested that the INSR showed a significant thermal stability-shifted effect upon AMA treatment. Other neurotrophic signaling pathways in PC12 cells were investigated using specific inhibitors, Western blotting and PC12(rasN17) and PC12(mtGAP) mutants. The inhibitors of the glucocorticoid receptor (GR), phospholipase C (PLC) and protein kinase C (PKC), Ras, Raf and mitogen-activated protein kinase (MEK) significantly reduced the neurite outgrowth induced by AMA in PC12 cells. Furthermore, the phosphorylation reactions of GR, PLC, PKC and an extracellular signal-regulated kinase (ERK) were significantly increased after inducing AMA and markedly decreased after treatment with the corresponding inhibitors. Collectively, these results suggested that AMA-induced neuritogenic activity in PC12 cells potentially depended on targeting the INSR and activating the downstream Ras/Raf/ERK and PI3K/AKT signaling pathways. In addition, the GR/PLC/PKC signaling pathway was found to be involved in the neurogenesis effect of AMA.

## 1. Introduction 

Alzheimer’s disease (AD) is a type of progressive neurodegenerative disease that accounts for 60–70% of dementia cases and its symptoms include an initial memory loss, later visual, language and cognitive disorders and a decline in the executive capacity in daily life [1]. The World Alzheimer Report 2019 states that over 50 million people are estimated to live with dementia worldwide and the number of patients will increase to 152 million by 2050. Additionally, the current yearly expenditure of dementia is estimated to reach USD 1 trillion, which will double by 2030 [2]. Currently, several drugs on the market such as tacrine, rivastigmine, huperzine A, donepezil, galantamine and memantine are used to treat AD. However, only the symptoms are mitigated and the efficacy of the drugs is not ideal, implying that a new strategy is needed for an effective AD treatment [3].

The nerve growth factor (NGF), the first recognized neurotrophic factor that plays a very important role in the survival, growth and maintenance of neuron cells, has become a drug candidate [4]. Nevertheless, with its high polarity and large molecule weight, the NGF cannot pass through the blood-brain barrier (BBB) and is difficult to apply as a drug [5]. This finding indicates that discovering a small molecule with an NGF-mimicking activity may be a potential alternative for AD treatment. 

Given the characteristic of exhibiting sympathetic neuron-like phenotypes under the stimulation of the NGF, the PC12 cell line, which is derived from rat pheochromocytoma cells, is widely used as a model to screen small molecules with NGF-mimicking activities [6]. In previous studies, under the guidance of a PC12 cell bioassay system, several small molecules with NGF-mimicking activities were isolated from traditional Chinese medicines (TCMs) such as *Gentiana rigescens* Franch, *Lindernia crustacean* and *Desmodium sambuense* and the mechanism of the action studies was also identified [7,8,9,10,11,12].

The genus *Gentiana* is a major group in the Gentianaceae family and its major constituents include iridoids and secoiridoids, which are responsible for various biological activities, and other important molecules such as essential oils, xanthones and terpenoids [13]. *G. rigescens* Franch (Jian Long Dan in Chinese), a well-known TCM that is widely distributed in the Yunnan Province, southwest China, is generally utilized for hepatitis, rheumatism, cholecystitis and inflammation treatment [14]. This TCM is praised with its anti-aging activity and cognition-improving effect in ‘Sheng Nong’s Herbal Classic’, a classic book on TCM material medica. In previous studies, gentisides A–K, which are 11 novel neuritogenic benzoate-type molecules, were isolated from *G. rigescens* and their mixture was confirmed to alleviate the impaired memory of an AD model [7,8,15]. 

**Figure 1:**
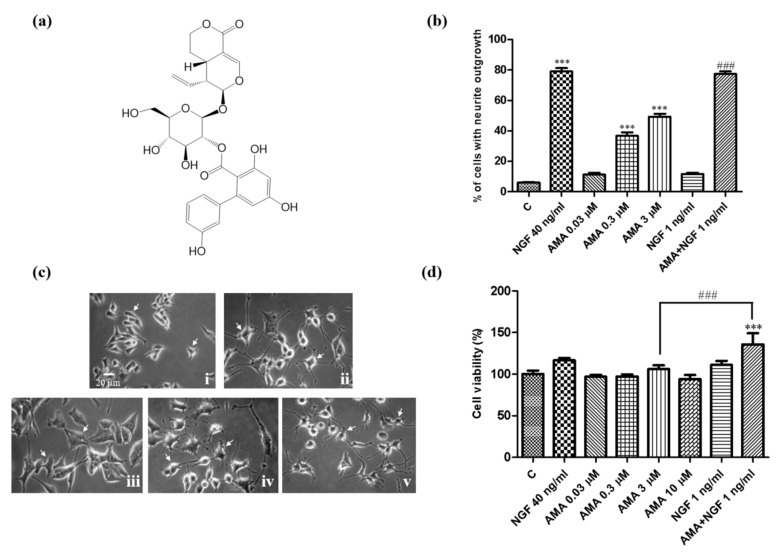
Neurogenesis effect of AMA in PC12 cells. (**a**) Chemical structure of AMA. (**b**) Percentage of PC12 cells with neurite outgrowth after treatment with AMA at different doses or AMA combined with a low dose of the NGF. (**c**) Morphological changes in PC12 cells under an inverted optical microscope at 48 h after treatment with (**i**) control (0.5% DMSO); (**ii**) NGF (40 ng/mL); (**iii**) NGF (1 ng/mL); (**iv**) AMA (3 μM); (**v**) AMA (3 μM) + NGF (1 ng/mL). (**d**) Cell viability analysis results of PC12 cells after treatment with various doses of AMA or AMA combined with the NGF. Each experiment was repeated three times. The data were expressed as a mean ± SEM. *** indicates significant differences at *p* < 0.001 compared with the negative control and ^###^ indicates a significant difference at *p* < 0.001 compared with the 3 μM AMA group.

In the present study, a secoiridoid-type compound was isolated from *G. rigescens* Franch. The chemical structure was determined as amarogentin (AMA) (Figure 1a). AMA was previously reported by our group to be a molecule with anti-aging and neuroprotection effects by an anti-oxidative stress activity [16]. Herein, the NGF-mimicking and NGF-enhancing activities of AMA were revealed and the mechanism of the action of the neurite outgrowth induced by AMA was investigated by using specific inhibitors in combination with Western blotting assays. Furthermore, the potential target was predicted using the cellular thermal shift assay (CETSA) and a small-interfering RNA (siRNA) analysis. The results indicated that AMA potentially targeted the insulin receptor and activated the PI3K/AKT and Ras/Raf/MEK/ERK signaling pathways. In addition, the GR/PLC/PKC was also involved in the neuritogenic activity of AMA in PC12 cells.

## 2. Experimental Section

### 2.1. Chemicals and Reagents

TrkA (k252a), GR (RU486), PI3K (LY294002), MEK/ERK (U0126) and PKC (Go6983) inhibitors, DMSO and NGF were purchased from Sigma—Aldrich Co. (St. Louis, MO, USA). The Ras inhibitor (farnesylthiosalicylic acid) was purchased from Cayman Chemical (Ann Arbor, MI, USA). The INSR (HNMPA-[AM]_3_), PLC (U73343) and Raf (AZ628) inhibitors were purchased from Santa Cruz Biotechnology (Dallas, TX, USA). The TrkB inhibitor (ANA-12) was purchased from Selleck (Shanghai, China) (see the details in Supplementary Table S1). Insulin and demethylasterriquinone B1 were purchased from YEASEN Biotech Co. Ltd. (Shanghai, China) and GlpBio Technology (Shanghai, China), respectively. 

### 2.2. Preparation of the AMA

AMA was isolated from the roots of *G. rigescens* and the chemical structure was determined by comparing the ^1^H NMR and ^13^C NMR spectra with the reported literature (Figure 1a). The detailed separation and structure elucidation steps were reported in a previous study [16].

### 2.3. Evaluation of the Neuritogenic Activity

The neuritogenic activity was evaluated as described in our previous paper [12]. Briefly, in each well of a 24-well microplate, around 50,000 PC12 cells were seeded and cultured under humidified conditions with 5% CO_2_ at 37 ℃ for 24 h. After 24 h, 1 mL of serum-free Dulbecco’s modified eagle medium (DMEM) containing a test sample or DMSO (0.5%) was used to replace the previous medium in each well. An NGF (40 ng/mL) was used as the positive control. Approximately 100 cells were counted thrice from a randomly selected area. Cells with a neurite outgrowth longer than the diameter of its body were counted as positive cells. The percentage of the positive cells in the selected area was regarded as the activities and the results were expressed as a mean ± SEM.

In the inhibitor test, the cells in each well of a 24-well microplate were first pretreated with 500 μL of the culture medium containing the specific inhibitor for 30 min. After this, 500 μL of the culture medium containing the sample or DMSO (0.5%) was added. The morphological changes in the cells were observed after 48 h. 

In addition, the wide-type of the PC12 cell lines and corresponding mutants (PC12(rasN17), PC12(mtGAP)) were provided by Prof. Hiroyuki Osada (RIKEN Center for Sustainable Resource Science, Japan).

### 2.4. Analysis of the Cell Viability by Using the MTT Assay

The cell viability was determined in accordance with the mitochondria-dependent reduction of MTT to purple formazan. Briefly, cells with AMA at concentrations of 0, 0.03, 0.3, 3 and 10 μM or AMA (3 μM) combined with a low dose of NGF were incubated for 48 h. The medium was removed carefully by aspiration. Afterward, 0.5 mL of fresh medium containing MTT (200 μg/mL) was added to each well and plates were incubated at 37 °C for 2 h. The medium in each well was then completely replaced with 0.2 mL DMSO to solubilize the formazan crystals. The resultant formazan was detected using a plate reader at 570 nm. All experiments were repeated at least three times.

### 2.5. Primary Culture of Mouse Cortical Neuron Cells

According to a previous study, primary cortical neuron cultures were prepared from the brains of C57BL/6J mice at embryonic day 17 [12]. Briefly, the cortex was digested in 0.5% trypsin in a 5% CO_2_ incubator at 37 °C for 20 min. Around 6×10^4^ neurons were seeded into the poly-L-lysine-coated 24-well plates in a serum-free neurobasal medium (Gibco, Grand Island, NY, USA). Samples with different concentrations (AMA at 0.1, 0.3, 1 and 3 μM; 0.1 μM AMA together with 1 ng/mL NGF) were added to each well and incubated for 24 h and 0.5% DMSO and NGF were used as negative and positive control samples, respectively. After 72 h of treatment with samples, 500 nM of NeuO were added to the cultures for 1 h. Fluorescence microscopy at an excitation/emission wavelength of 430/560 nm was then used to image the neurons. Image J software (National Institutes of Health, Bethesda, MD, USA) was used to measure the relative length of the neurite outgrowth of the neurons. The results were expressed as a mean ± SEM.

### 2.6. Western Blot Analysis

A Western blot analysis was performed in accordance with previous studies [12]. Briefly, in each 60 mm culture dish containing 5 mL DMEM, approximately 2 × 10^6^ PC12 cells were seeded and incubated for 24 h. For the time-dependent study of AMA, AMA (3 μM) was supplemented to the dishes, which were incubated for specific time periods. For the study of the inhibitors, AMA (3 μM) or AMA (3 μM) with a low dose of the NGF (1 ng/mL) were added to the dishes, which were then incubated for a certain period (2 h for GR, p-GR, PLC and p-PLC; 8 h for the INSR and p-INSR; 24 h for AKT and p-AKT; 48 h for ERK1/2, p-ERK1/2, PKC and p-PKC). Sodium dodecyl sulphate polyacrylamide gel electrophoresis was used to separate the proteins (15 μg) and transfer them onto a PVDF membrane. The membranes were incubated with primary antibodies and secondary antibodies (see the details in Supplementary Table S2). The antigens were visualized using a high sensitivity chemiluminescence detection kit (Beijing Cowin Biotech Company, Beijing, China). The primary antibodies used for immunoblotting were as follows: anti-insulin receptor antibody, anti-phospho-insulin receptor (Tyr1150/1151) antibody, anti-phospho-AKT (Ser473), anti-AKT antibody, anti-phospho-p44/42 MAPK (ERK1/2) (Thr202/Tyr204) antibody, anti-44/42 MAPK (ERK1/2) antibody, anti-phospho-PLC γ antibody, anti-PLC antibody, anti-phospho-PKC antibody, anti-PKC antibody (Cell Signaling Technology, Boston, MA, USA), anti-phospho-GR antibody (Affinity BioReagents, OH, USA) and anti-GR antibody (Santa Cruz, CA, USA) and GAPDH antibody (Beijing Cowin Biotech Company, Beijing, China). The secondary antibodies used in this study were as follows: horseradish peroxidase-linked anti-rabbit and anti-mouse IgGs (Beijing Cowin Biotech Company, Beijing, China). The bands were quantitatively measured using ImageJ software (National Institutes of Health, Bethesda, MD, USA).

### 2.7. Cellular Thermal Shift Assay

A CETSA was performed as described in other reports [17]. First, in 60 mm dishes containing 5 mL DMEM, 2 × 10^6^ cells were separately added and incubated for 24 h. In each plate, AMA was added at a final concentration of 3 μM. After a continuous incubation for 8 h, cells were collected and heated at temperatures ranging from 46 °C to 66 °C. Finally, a Western blot analysis was used to detect the changes in the INSR protein and GR protein.

### 2.8. RNA Interference

PC12 cells were transfected with different concentrations of FAM-labelled siRNA to evaluate the transfection efficiency. Finally, 150 nM was decided as the final concentration to perform the experiment at which 90% of the transfection efficiency was obtained. The following primer sequences were used to generate siRNAs that knocked down the INSR and the negative control (Sangon Biotech Co. Ltd., Shanghai, China): for INSR-4295, sense: 5′-GUG AAG AGC UGG AGA UGG ATT-3′, anti-sense: 5′-UCC AUC UCC AGC UCU UCA CTT-3′; for the negative control, sense: 5′-UUC UCC GAA CGU GUC ACG UTT-3′, anti-sense: 5′-ACG UGA CAC GUU CGG AGA ATT-3′. 

The transfection of PC12 cells with an siRNA was performed on the basis of the manufacturer’s instructions. Briefly, in each well of 24-well plates, 5 × 10^4^ cells were seeded and allowed to reach 70–90% confluence in a growth medium without antibiotics one day before the transfection. SiRNA against the INSR or the negative control siRNA were then used at a concentration of 150 nM with Lipofectamine 2000 (Invitrogen) as the transfection agent. After 6 h of transfection, the fresh medium containing 3 μM AMA or AMA combined with a low dose of the NGF was used to replace the previous medium in the plates and the plate was then incubated for another 24 h. The cell morphological features were observed and recorded using an inverted microscope fitted with a camera. The percentage of the cells with a neurite outgrowth was expressed as the mean ± SEM. Finally, a Western blot analysis was used to detect the changes in the INSR protein.

### 2.9. Statistical Analysis

Data were presented as a mean ± SEM of three independent experiments in triplicate. Data were subjected to a one-way ANOVA and a Tukey’s post hoc analysis by using the GraphPad Prism software. *p* < 0.05 was considered statistically significant.

## 3. Results

### 3.1. AMA-Induced Neuritogenic Effect in PC12 Cells and in Primary Cortical Neuron Cells

The neuritogenic activity of AMA was first detected in PC12 cells. PC12 cells were treated with different concentrations of AMA (0.03, 0.3 and 3 μM) for 48 h. The results showed that AMA induced neurite outgrowth in PC12 cells in a dose-dependent manner. The percentages of the cells with a neurite outgrowth after treatment with 0, 0.03, 0.3 and 3 μM of AMA were 6.0% ± 0.6%, 11.3% ± 1.2%, 36.7% ± 2.3% (*p* < 0.001) and 53.0% ± 2.1% (*p* < 0.001), respectively (Figure 1b). Interestingly, AMA with a low dose of the NGF (1 ng/mL) significantly increased the percentage of PC12 cells with neurite outgrowth from 53.0% ± 2.1% to 77.3% ± 1.3% (*p* < 0.001, Figure 1b). The morphological changes in PC12 cells after treatment with 3 μM of AMA and AMA combined with 1 ng/mL of NGF are displayed in Figure 1c. These results indicated that AMA exhibited NGF-mimicking and NGF-enhancing activities in PC12 cells. The effect of AMA in PC12 cell viability was then determined using the 3-(4,5-dimethylthiazol-2-yl)-2,5-diphenyl tetrazolium bromide (MTT) analysis. The viabilities of PC12 cells were 96.9% ± 2.3%, 97.3% ± 2.4%, 106.1% ± 4.4% and 94.0% ± 4.9% after treatment with AMA at doses of 0.03, 0.3, 3 and 10 μM, respectively (Figure 1d). None of these concentrations produced considerable cytotoxicity as detected by the MTT assay. Furthermore, the viability of PC12 cells in the 3 μM AMA-treated group was significantly increased to 135.7% ± 13.4% after adding a low dose of the NGF (1 ng/mL, *p* < 0.001, Figure 1d). These results suggested that AMA showed no cytotoxicity at a dose of 10 μM and that the low-dose NGF could increase the cell viability of AMA in PC12 cells. 

**Figure 2:**
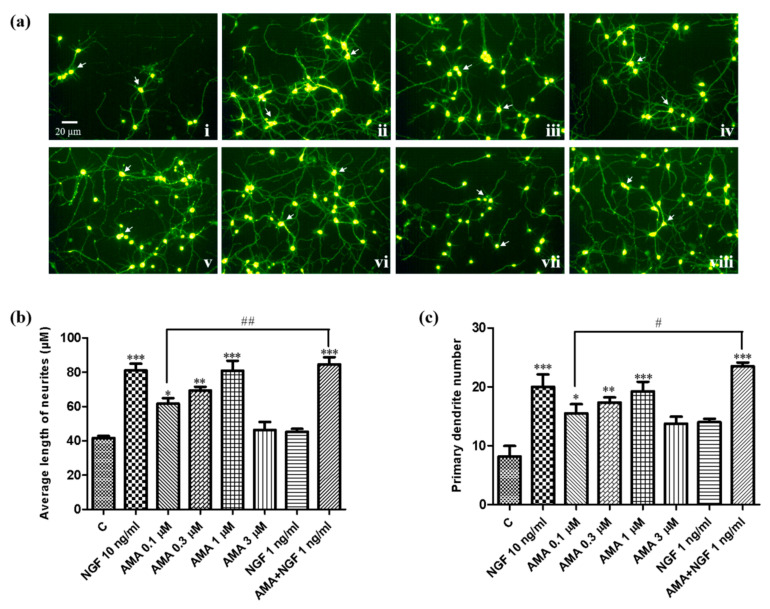
Neurogenesis effect of AMA in primary cortical neuron cells. (**a**) Micrographs of primary cortical neuron cells at 48 h after treatment with (**i**) control (0.5% DMSO); (**ii**) NGF (10 ng/mL); (**iii**) NGF (1 ng/mL); (**iv**) AMA (0.1 μM); (**v**) AMA (0.3 μM); (**vi**) AMA (1 μM); (**vii**) AMA (3 μM); (**viii**) AMA (0.1 μM) + NGF (1 ng/mL). (**b**) Average length of neurite outgrowth of the indicated groups in the primary cortical neuron cells. (**c**) Average primary dendrite number in each group. Each experiment was repeated three times. The data were expressed as a mean ± SEM. *, ** and *** indicate significant differences at *p* < 0.05, *p* < 0.01 and *p* < 0.001 compared with the negative control; ^#, ##^ indicate a significant difference at *p* < 0.05 and *p* < 0.01 compared with the 0.1 μM AMA group.

In addition, the neuritogenic effect of AMA was further estimated in the primary cortical neuron cells. As shown in Figure 2, the neurite outgrowth was increased significantly after treatment with different concentrations of AMA and AMA with the NGF. The morphological changes in the primary cortical neurons are shown in Figure 2a. The average of the neurite length and primary dendrite number are displayed in Figure 2b,c, respectively. Treatment with AMA at 0.1, 0.3 and 1 μM significantly increased the neurite length from 41.7 ± 1.2 μm to 61.7 ± 3.2 μm (*p* < 0.05), 69.3 ± 2.2 μm (*p* < 0.01) and 80.9 ± 5.7 μm (*p* < 0.001), respectively. Moreover, 0.1 μM AMA combined with 1 ng/mL of NGF increased the neurite length to a level that was comparable with the effect of the NGF at 10 ng/mL (*p* < 0.01). Collectively, these results demonstrated that AMA exhibited significant neuritogenic activity in PC12 cells and in primary cortical neuron cells.

### 3.2. Effect of AMA on the Ras/Raf/MEK/ERK Signaling Pathway

**Figure 3:**
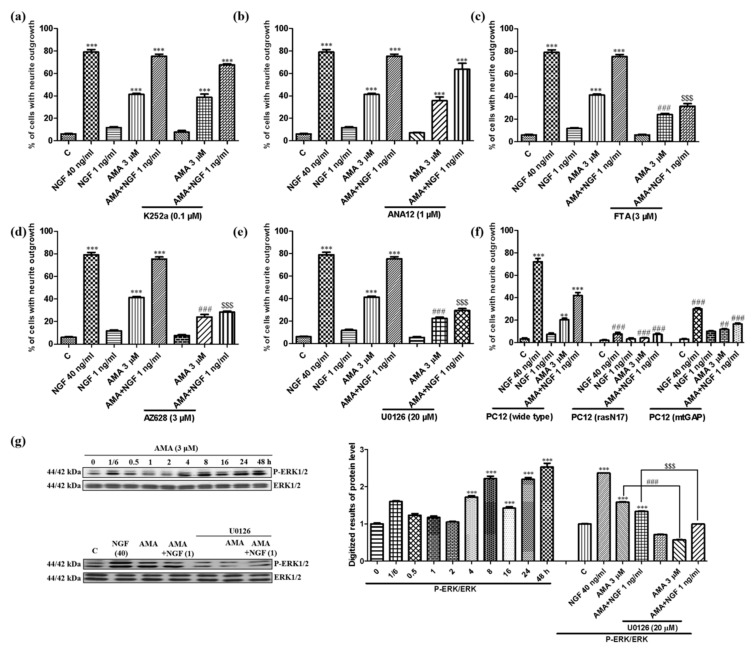
Effect of AMA on the Ras/Raf/MEK/ERK signaling pathway in PC12 cells. (**a**,**b**) Effect of TrkA inhibitor K252a and TrkB inhibitor ANA-12 on the neurite outgrowth induced by AMA and AMA combined with the NGF. (**c**–**e**) Effects of Ras, Raf and MEK inhibitors on the neurogenesis activity of AMA and AMA combined with the NGF. (**f**) Percentage of the neurite outgrowth induced by AMA and AMA combined with the NGF for 48 h in wide-type or Ras mutant PC12 cells. (**g**) Phosphorylation of ERK at different time points induced by AMA. The ERK phosphorylation was reduced by the inhibitor of MEK and quantified using Western blots through ImageJ software. Each experiment was repeated three times. ** and *** indicate significant differences at *p* < 0.01 and *p* < 0.001 compared with the negative control; ^##, ###^ indicate a significant difference at *p* < 0.01 and *p* < 0.001 compared with the 3 μM AMA group; ^$$$^ indicates a significant difference at *p* < 0.001 compared with the AMA-combined NGF group.

Different neurotrophic factors such as NGF and BDNF specifically bind to the transmembrane receptors TrkA and TrkB and activate several kinases to stimulate the function of differentiation and survival in neuron cells [18,19]. Therefore, the mechanism of the action of AMA was first investigated using the inhibitors of TrkA and TrkB. However, the neurite outgrowth induced by AMA or AMA combined with the NGF did not change after the treatment with the inhibitor of TrkA, K252a (Figure 3a). Similarly, the inhibitor of TrkB, ANA-12, did not affect the NGF-mimicking or NGF-enhancing effect of AMA in PC12 cells (Figure 3b).

Ras/Raf//MEK/ERK was believed to be the major cascade for the NGF-stimulated differentiation in PC12 cells [20]. Therefore, the effect of these signaling pathways was investigated using specific inhibitors, mutants and a Western blot analysis. As displayed in Figure 3c–e, after adding the inhibitors of Ras (farnesylthiosalicylic acid, FTA), Raf (AZ628) and MEK (U0126), the neurite outgrowth induced by AMA was significantly reduced from 53.0% ± 2.1% to 24.0% ± 1.2% (*p* < 0.001), 22.3% ± 1.2% (*p* < 0.001) and 21.0% ± 1.0% (*p* < 0.001), respectively. Similarly, the neuritogenic activity of AMA combined with the NGF was decreased by these above mentioned inhibitors from 75.3% ± 1.9% to 31.3% ± 2.4% (*p* < 0.001), 28.3% ± 0.9% (*p* < 0.001) and 29.3% ± 1.7% (*p* < 0.001), respectively (Figure 3c–e). 

Furthermore, the Ras mutant types of PC12 cells including the membrane-targeted PC12(mtGAP) or the dominant inhibitory mutant PC12(rasN17) were used to detect the effect of AMA on the Ras protein. AMA or AMA combined with the NGF failed to induce the neurite outgrowth on the Ras mutant cell lines due to the inhibition of the Ras function. This finding suggested that the Ras signaling was involved in the effect of AMA (Figure 3f, Supplementary Figure S1).

The effect of AMA on ERK phosphorylation at the protein level was studied. The phosphorylation of ERK was increased from 4 h and peaked at 48 h (Figure 3g, Supplementary Figure S2). Meanwhile, the ERK phosphorylation in the AMA-treated group or the AMA with a low dose of NGF-treated group was diminished by the inhibitor of MEK, U0126 (Figure 3g). These results indicated that TrkA and TrkB were not involved in the neurogenesis effect of AMA. However, the Ras/Raf/MEK/ERK signaling pathway took an important role in the neurogenesis effect of AMA. 

### 3.3. Effect of AMA on the INSR/PI3K/AKT Signaling Pathway

**Figure 4:**
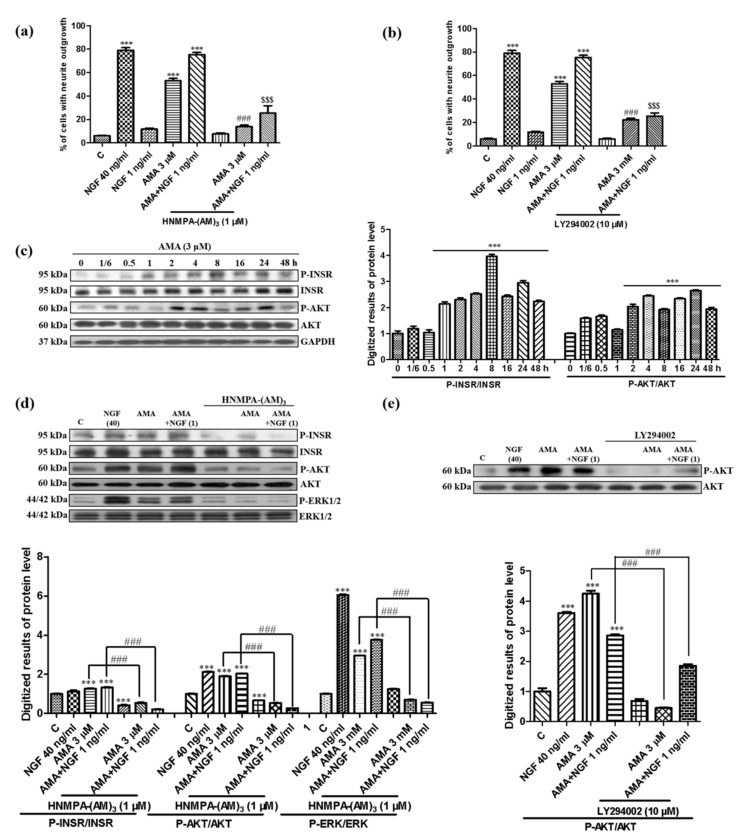
Effect of amarogentin on the insulin receptor/PI3K/AKT signaling pathway in PC12 cells. (**a**,**b**) Effect of the insulin receptor inhibitor HNMPA-(AM)_3_ and PI3K inhibitor LY294002 on the neurite outgrowth induced by AMA and AMA combined with the NGF. (**c**) AMA-induced phosphorylation of the insulin receptor and AKT in a time-dependent manner and quantification of the Western blots by using ImageJ software. (**d**) Phosphorylation of the insulin receptor, AKT and ERK induced by AMA or AMA combined with the NGF was decreased by the inhibitor HNMPA-(AM)_3_. (**e**) Phosphorylation of AKT induced by AMA or AMA combined with the NGF was decreased by the inhibitor LY294002. Each experiment was repeated three times. *** indicates significant differences at *p* < 0.001 compared with the negative control; ^###^ indicates a significant difference at *p* < 0.001 compared with the 3 μM AMA group and ^$$$^ indicates a significant difference at *p* < 0.001 compared with the AMA-combined NGF group.

Growing evidence shows that insulin plays an important role in brain functions such as cognitive and memory improvement. Insulin binds to the INSR and activates the PI3K/AKT pathway, thereby enhancing the cell growth and survival [21]. Therefore, the inhibitor of the INSR, HNMPA-(AM)_3_, was used to study the mechanism of the action of AMA. After treatment with HNMPA-(AM)_3_, the AMA-induced neurite outgrowth was significantly decreased from 53.0% ± 2.1% to 11.7% ± 0.9% (*p* < 0.001) (Figure 4a). Moreover, the neurite outgrowth induced by AMA was reduced after treatment with the inhibitor of PI3K, LY294002, from 53.0% ± 2.1% to 22.3% ± 1.2% (*p* < 0.001) (Figure 4b). The neurite outgrowth of PC12 cells induced by AMA combined with the NGF was also decreased by HNMPA-(AM)_3_ and LY294002 (Figure 4a,b). 

Subsequently, the phosphorylation of the INSR and AKT induced by AMA were investigated in a time-dependent manner. The INSR phosphorylation increased at 1 h and peaked at 8 h after treatment with AMA (Figure 4c, Supplementary Figure S3). Furthermore, the phosphorylation of AKT after the treatment with AMA was increased at 2 h and peaked at 24 h (Figure 4c). The increase in the phosphorylation of the INSR and downstream protein AKT and ERK in the AMA with or without 1 ng/mL of NGF were significantly decreased by HNMPA-(AM)_3_ (Figure 4d, Supplementary Figure S3). In addition, the phosphorylation of AKT induced by AMA and AMA combined with the NGF were also reduced by the inhibitor of PI3K, LY294002 (Figure 4e, Supplementary Figure S3). These results suggested that the INSR/PI3K/AKT signaling pathway exerted an important effect on the AMA-induced neurite outgrowth in PC12 cells.

### 3.4. Effect of AMA on the GR/PLC/PKC Signaling Pathway

**Figure 5:**
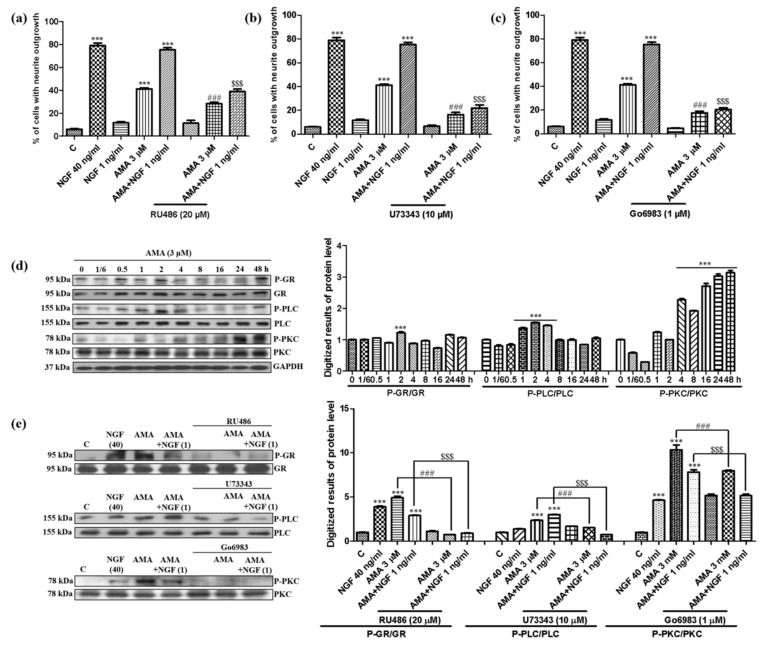
Effect of AMA on the GR/PLC/PKC signaling pathway in PC12 cells. (**a**–**c**) Effect of GR (RU486), PLC (U73343) and PKC (Go6983) inhibitors on the neurite outgrowth induced by AMA and AMA combined with the NGF. (**d**) AMA-stimulated phosphorylation of GR, PLC and PKC proteins in a time-dependent manner and the quantification of Western blots by using ImageJ software. (**e**) Phosphorylation of GR, PLC and PKC induced by AMA or AMA combined with the NGF reduced by the corresponding inhibitors and quantified using Western blots through ImageJ software. Each experiment was repeated three times. *** indicates significant differences at *p* < 0.001 compared with the negative control; ^###^ indicates a significant difference at *p* < 0.001 compared with the 3 μM AMA group and ^$$$^ indicates a significant difference at *p* < 0.001 compared with the AMA-combined NGF group.

GR has been reported to regulate a series of genes important for neuronal structure and plasticity and is involved in the neuritogenic activity in PC12 cells [22,23]. Therefore, the inhibitor of GR, RU486, was used to elucidate the mechanism of the action of AMA. The percentage of cells with a neurite outgrowth was significantly decreased from 53.0% ± 2.1% to 28.3% ± 1.2% (*p* < 0.001) after treatment with RU486 (Figure 5a). Given that the PLC/PKC signaling pathway is located at the downstream of GR and plays an important role in cell survival and differentiation [24], the inhibitors of PLC (U73343) and PKC (Go6983) were used to examine the effect of AMA. The neurite outgrowth of AMA was diminished from 53.0% ± 2.1% to 16.3% ± 2.0% and 17.7% ± 1.6% (*p* < 0.001) after the addition of U73343 and Go6983, respectively (Figure 5b,c). Similarly, the effect of AMA combined with a low dose of the NGF was also inhibited by RU486, U73343 and Go6983 (Figure 5a–c). 

The phosphorylation of GR/PLC/PKC was then determined at the protein level by using a Western blot analysis. The GR phosphorylation peaked at 2 h and was reduced by RU486 (Figure 5d,e, Supplementary Figure S4). The PLC phosphorylation was increased from 1 h, peaked at 2 h and decreased by the inhibitor of PLC, U73343 (Figure 5d,e). Furthermore, the phosphorylation of PKC peaked at 48 h and was reduced by Go6983, the inhibitor of PKC (Figure 5d,e). The AMA combined with the NGF group changed in a similar way (Figure 5d,e). These results demonstrated that the AMA-induced neuritogenic activity in PC12 cells was related to the GR/PLC/PKC signaling pathway.

### 3.5. Identification of the Target Protein for AMA by Using siRNA Analysis and CETSA

**Figure 6:**
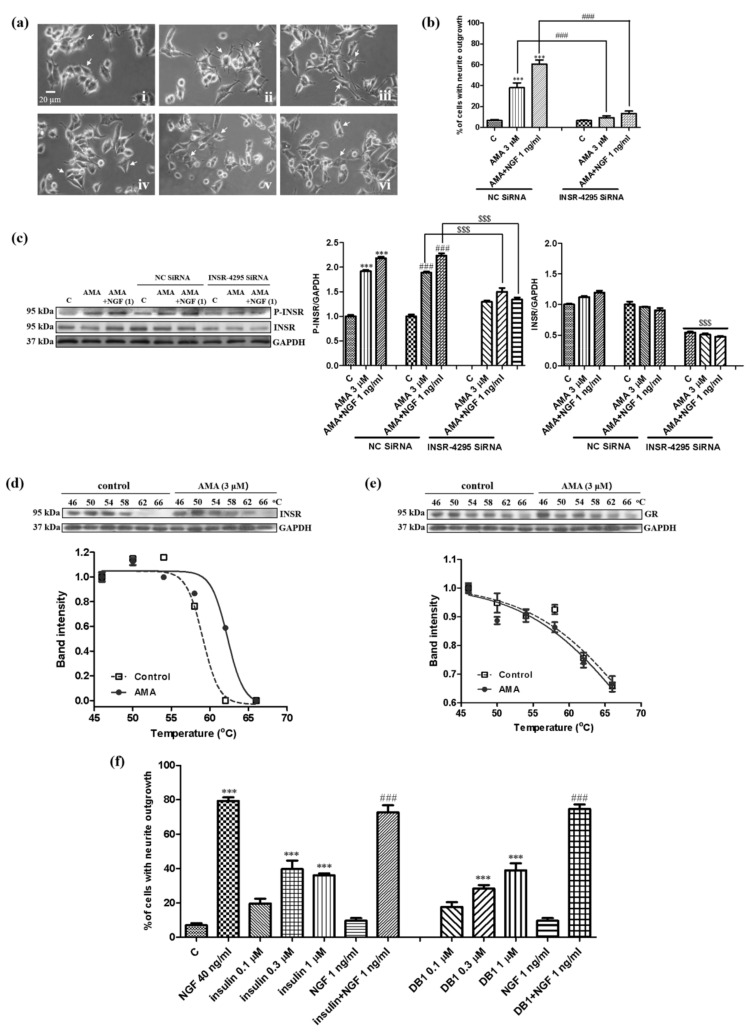
Target prediction of AMA in PC12 cells by using siRNA and a CETSA assay. (**a**) Microphotographs of PC12 cells after treatment with siRNA and AMA or AMA combined with the NGF: (**i**) negative control siRNA, control (0.5% DMSO); (**ii**) negative control siRNA, AMA (3 μM); (**iii**) negative control siRNA, AMA (3 μM) + NGF (1 ng/mL); (**iv**) insulin receptor siRNA, control (0.5% DMSO); (**v**) insulin receptor siRNA, AMA (3 μM); (**vi**) insulin receptor siRNA, AMA (3 μM) + NGF (1 ng/mL). (**b**) Percentage of cells with a neurite outgrowth after treatment with siRNA and AMA or AMA combined with the NGF. (**c**) Western blot analysis for the insulin receptor after transfection with negative siRNA or insulin receptor siRNA and treatment with AMA or AMA combined with the NGF. Cells were transfected with Lipofectamine 2000 and 150 nM siRNA for 6 h and treated with AMA or AMA combined with the NGF. (**d**,**e**) CETSA of PC12 cells on the insulin receptor or GR protein and corresponding fitting curves. (**f**) Neuritogenic activity of insulin and demethylasterriquinone B1 in PC12 cells. ^***^, ^###^ and ^$$$^ indicate significant differences at *p* < 0.001 compared with the corresponding groups.

Considering that TrkA and TrkB are not involved in the neurogenesis effect of AMA, we predicted that the INSR or GR protein might be the potential target of AMA. Given that the inhibition effect of the INSR for AMA was stronger than that of GR, the INSR was first considered as the potential target of AMA. The 5-carboxyfluorescein (FAM)-labelled siRNA was initially used to confirm the optimal transfection concentration of siRNA and whether AMA targeted the INSR. Approximately 90% of the PC12 cells produced fluorescence after treatment with 150 nM of the FAM-labelled siRNA and 150 nM of the INSR siRNA was used to perform the transfection (Supplementary Figure S5). The INSR siRNA was transfected into PC12 cells for 6 h and treated with 3 μM AMA or AMA combined with the NGF. After the treatment of the PC12 cells with the INSR siRNA, the percentage of cells with a neurite outgrowth induced by AMA with or without a low dose of the NGF for 48 h was significantly decreased (Figure 6a,b). In addition, the total and the phosphorylation protein levels of the INSR were significantly decreased by the treatment with the INSR siRNA regardless of the AMA treatment (*p* < 0.001, Figure 6c, Supplementary Figure S6). Hence, these results indicated that the INSR might be the target protein of AMA. 

A CETSA was used to discover the target protein of molecules on the basis of the thermal stabilization of proteins upon ligand binding [17]. Therefore, a CETSA was used to detect the binding correlations between the INSR and AMA to further confirm the potential target of AMA. After treating the PC12 cells with dimethyl sulfoxide (DMSO) or AMA and heating at temperature ranging from 46 °C to 66 °C, the immunoblotting analysis was conducted using a specific antibody for the INSR. The results suggested a significant thermal stabilization of the INSR protein upon AMA treatment (Figure 6d, Supplementary Figure S6). At the same time, the change of GR at the protein level was detected using the same method. As expected, the GR protein did not show the thermal stability-shifted effect after the AMA treatment (Figure 6e, Supplementary Figure S6). Furthermore, other known insulin agonists such as insulin and demethylasterriquinone B1 (DB1) were selected to detect whether they exhibited a similar neurogenesis as AMA in PC12 cells [25]. The results indicated that both showed a significant NGF-mimicking and NGF-enhancing activity in the PC12 cells (Figure 6f, Supplementary Figure S7). These results indicated that AMA might target the INSR to produce the NGF-mimicking activity.

## 4. Discussion

Aging is a major risk factor for age-related diseases such as Parkinson’s disease and AD [26]. We speculated that if we prevent or delay aging, we can prevent the occurrence of AD or cure AD. Our laboratory began to screen small anti-aging molecules from food and TCMs ten years ago to verify this hypothesis. To date, we have found more than 30 anti-aging compounds with different types of chemical structures such as sterols, benzoquinones, phenols and terpenes [27,28,29,30]. Furthermore, we have indicated that cucurbitacin B with an anti-aging effect can improve the memory of APP/PS1 mice via the target cofilin and the regulation of GR signaling pathways [23,31]. These results indicate that anti-aging substances may prevent and treat AD.

*G. rigescens* Franch is a TCM used to treat hepatitis, rheumatism, cholecystitis and inflammation in China [14]. In our previous study, we discovered gentisides A–K with a novel NGF-mimicking effect from the nonpolar extract of this plant and indicated that a mixture of benzoates could alleviate the impaired memory of AD model mice induced by scopolamine [15]. We have also focused on the water layer of *G. rigescens* Franch to isolate active molecules under the guidance of PC12 cells and a yeast replicative lifespan assay to understand whether the small molecules of the polar part have the same function. We have found that AMA produces anti-aging effects on yeasts and neuron protection in PC12 cells via anti-oxidative stress [16]. In the present study, we used PC12 cells and primary cortical neuron cells to investigate the neurogenesis effect of AMA. The morphological changes of PC12 cells and primary cortical neuron cells after AMA treatment suggested that AMA had a neurogenesis effect on PC12 cells and primary cortical neuron cells (Figure 1 and Figure 2). These results were consistent with those of our previous reports [12,23].

The target protein identification has an important role in drug development and can provide strong evidence for the elucidation of the mechanism of the action, safety evaluation and targeted treatment of a disease [32]. Therefore, we first focused on the target protein discovery of AMA to perform deep research with specific inhibitors, siRNA, a CETSA and a Western blot analysis. The results of the specific inhibitors for TrkA, TrkB, INSR, GR, PI3K, PLC, PKC and MEK and the Western blot analysis in Figure 3, Figure 4, Figure 5 and Figure 6 indicated that AMA induced neuritogenic activity in PC12 cells by activating the INSR and regulating the PI3K/AKT/Ras/Raf/ERK and GR/PLC/PKC signaling pathways. Interestingly, the mechanism of the action of AMA for its NGF-mimicking effect was different from that of previously reported compounds (such as ABG-001, lindersin B, 3beta,23,28-trihydroxy-12-oleanene 3beta-caffeate and CuB). Tetradecyl 2,3-dihydroxybenzoate (ABG-001) was designed and synthesized as a lead compound in accordance with the gentiside series to induce neurogenesis in PC12 cells by the IGF-1R/PI3K/MAPK signaling pathway [9,10]. Lindersin B from *L. crustacea* induced neuritogenic activity through the activation of the TrkA/PI3K/ERK signaling pathway [11]. 3beta,23,28-trihydroxy-12-oleanene 3beta-caffeate from *D. sambuense* induced neurogenesis in PC12 cells mediated by the ER stress and BDNF-TrkB signaling pathways [12] and CuB induced neuritogenic activity by targeting cofilin and regulating the GR TrkA signaling pathways [23]. These molecules possess different structures but exhibit neurogenesis effects by activating various related signaling pathways. AMA was the first compound we discovered to target the INSR for the NGF-mimicking activity in PC12 cells. These results provided insights into the combination that the use of these molecules may have in increasing the therapy effect for AD.

TrkA and TrkB are specific transmembrane receptors that bind to neurotrophic factors such as NGF and BDNF [18,19]. Therefore, the effects of these two proteins were investigated. We found that TrkA and TrkB were not involved in the neurogenesis effect of AMA (Figure 3b). We focused on the INSR and GR to determine the target protein. The results of the INSR knockdown experiment, a CETSA and a Western blot analysis for the INSR and GR in Figure 6 revealed that the INSR was the potential target protein of AMA. Furthermore, known insulin agonists including insulin and DB1 showed similar neurogenesis effects as AMA in PC12 cells, which confirmed the INSR as the potential target of AMA (Figure 6). It was different from the target proteins of CuB, cofilin and 3beta,23,28-trihydroxy-12-oleanene 3beta-caffeate and ER stress [11,23]. AMA may have effects for diabetes and inflammation because of the involvement of insulin and GR signaling pathways [33,34].

**Figure 7:**
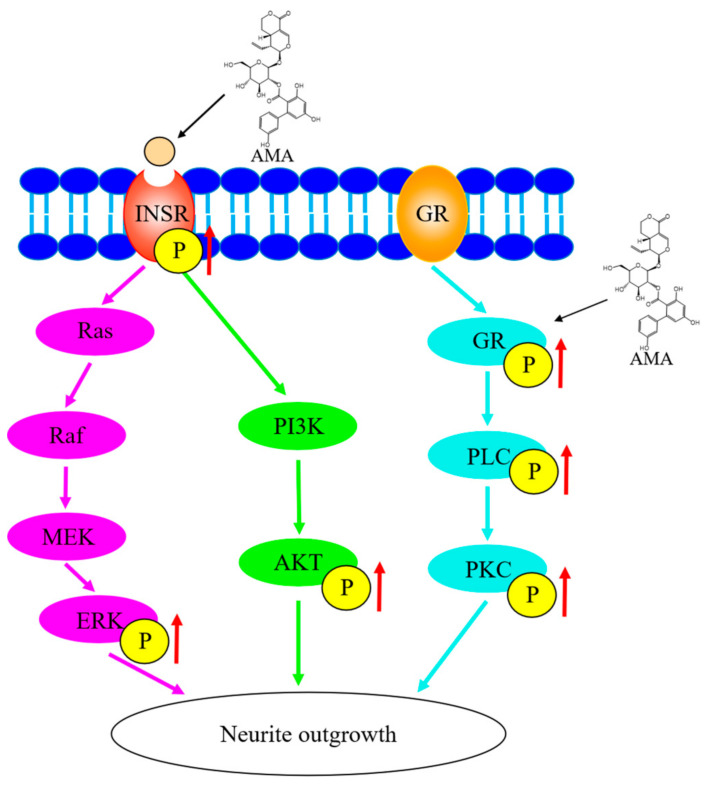
Proposed mechanism of the action of AMA in the neuritogenic activity in PC12 cells.

In conclusion, AMA from *Gentiana rigescens* Franch showed significant neuritogenic activity in PC12 cells and in primary cortical neuron cells. The neuritogenic activity induced by AMA in PC12 cells was through the targeting of the INSR and the regulation of the PI3K/AKT/Ras/Raf/ERK and GR/PLC/PKC signaling pathways (Figure 7). This study indicated the potential applications of AMA for its neurogenesis effect and provided evidence for the treatment of neurodegenerative diseases and anti-aging. Furthermore, the structure-activity relationship of AMA should be studied to discover the novel leading compounds and elucidate the underlying mechanism in animal levels and also applied to clinical trials. 

## Data Availability

All figures and data used to support this study are included within this article.

## Supplementary Materials

The following are available online at https://www.mdpi.com/article/10.3390/biomedicines9050581/s1, Figure S1: Differentiation of PC12 cells expressing dominant negative Ras (PC12(rasN17)) or membrane-targeted GAP (PC12(mtGAP)) induced by AMA or AMA combined with NGF, Figure S2: Origin data of Western blot analysis in Figure 3g, Figure S3: Origin data of Western blot analysis in Figure 4c–e, Figure S4: Origin data of Western blot analysis in Figure 5d,e, Figure S5: Microphotograph of PC12 cells after transfection with different concentrations of FAM-siRNA (50 nM, 100 nM and 150 nM), Figure S6: Origin data of Western blot analysis in Figure 6c–e, Figure S7: Microphotograph of PC12 cells after treatment with insulin and Demethylasterriquinone B1, Table S1: List of inhibitors used in this study, Table S2: List of antibodies used in this study.

## Abbreviations

Alzheimer’s disease (AD); amarogentin (AMA); blood-brain barrier (BBB); cellular thermal shift assay (CETSA); demethylasterriquinone B1 (DB1); dimethyl sulfoxide (DMSO); extracellular signal-regulated kinase (ERK); glucocorticoid receptor (GR); insulin receptor (INSR); mitogen-activated protein kinase (MEK); nerve growth factor (NGF); phospholipase C (PLC); protein kinase B (AKT); protein kinase C (PKC); small-interfering RNA (siRNA); traditional Chinese medicines (TCMs).
