# Supplementary material for: The Insulin Receptor: A Potential Target of Amarogentin Isolated from Gentiana rigescens Franch That Induces Neurogenesis in PC12 Cells
Source: Biomedicines. 2021 May 20;9(5):581. doi: 10.3390/biomedicines9050581 (PMC8160887; doi:10.3390/biomedicines9050581)
Supplement: Supplementary file 1 [file biomedicines-09-00581-s001.zip › biomedicines-1193961-supplementary.pdf]

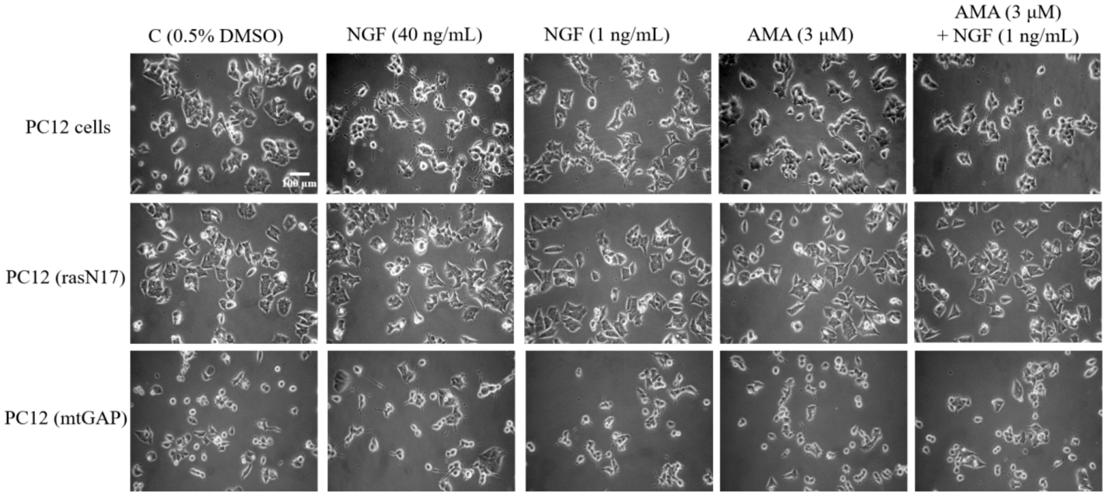

Supplementary Figure 1. Differentiation of PC12 cells expressing dominant negative Ras (PC12(rasN17)) or membrane-targeted GAP (PC12(mtGAP)) induced by AMA or AMA combined with NGF.

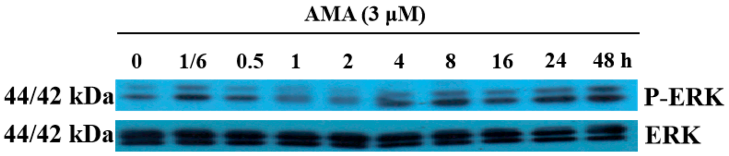

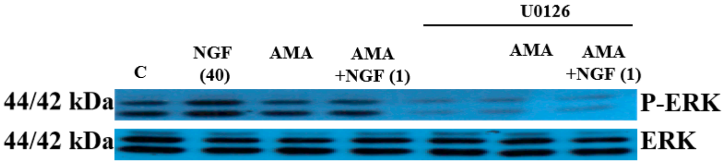

Supplementary Figure 2. Origin data of Western blot analysis in Figure 3g.

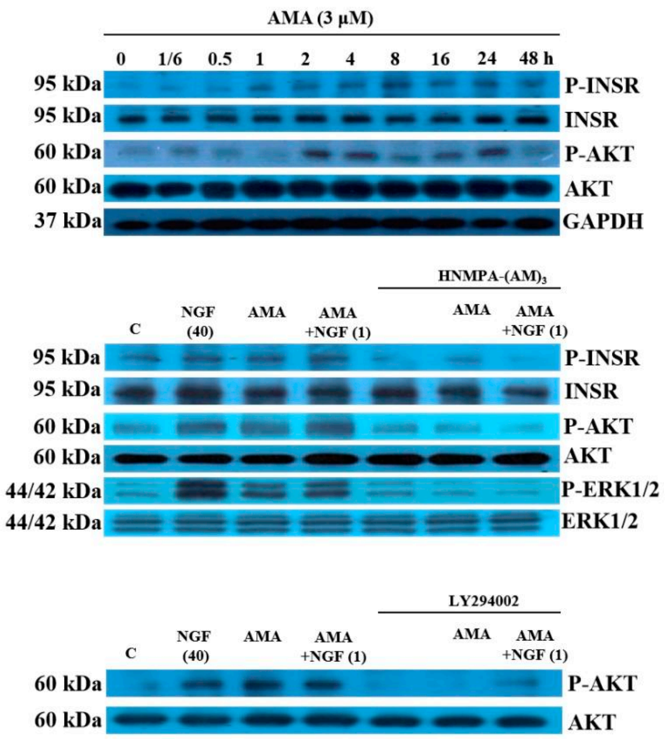

Supplementary Figure 3. Origin data of Western blot analysis in Figure 4c, 4d and 4e.

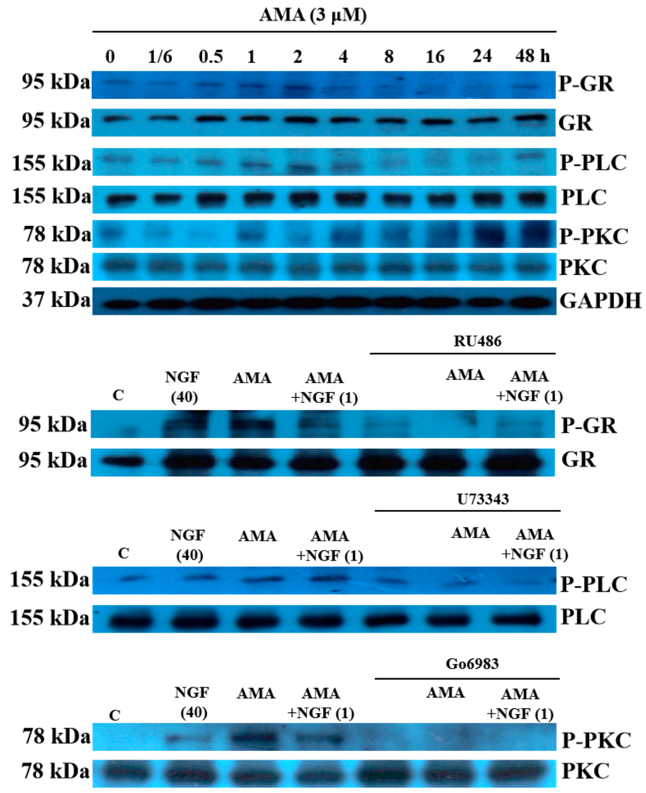

Supplementary Figure 4. Origin data of Western blot analysis in Figure 5d and 5e.

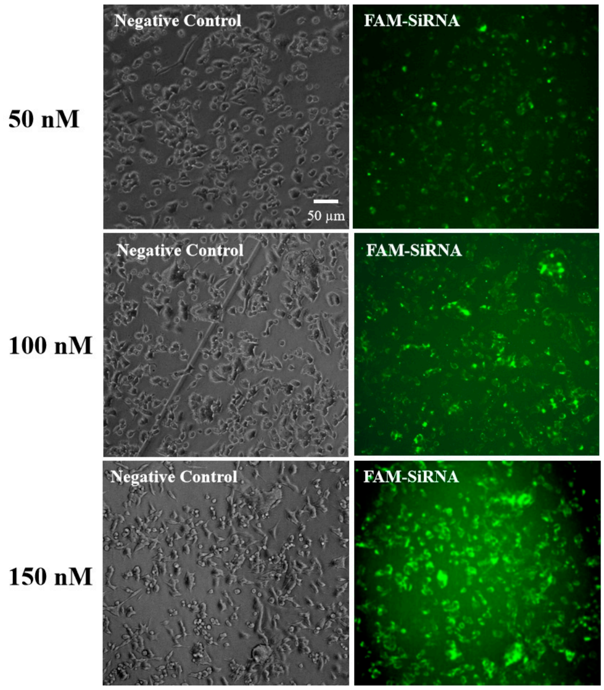

Supplementary Figure 5. Microphotograph of PC12 cells after transfection with different concentrations of FAM-siRNA (50 nM, 100 nM and 150 nM).

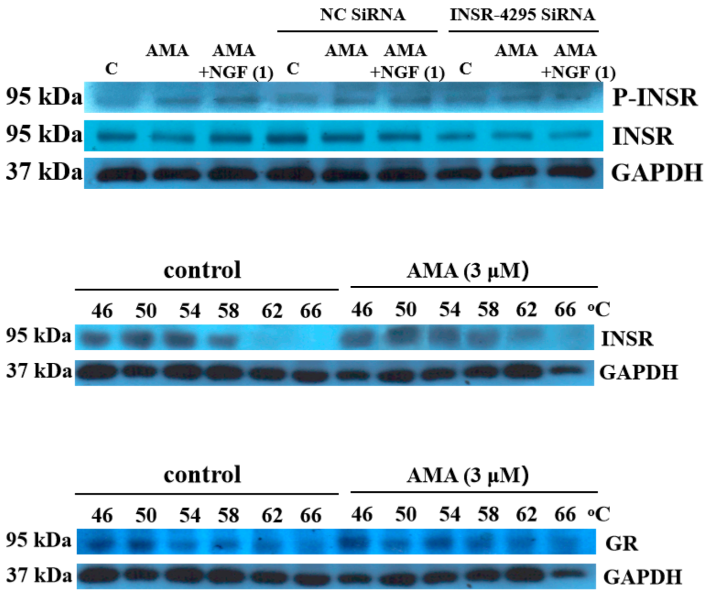

Supplementary Figure 6. Origin data of Western blot analysis in Figure 6c, 6d and 6e.

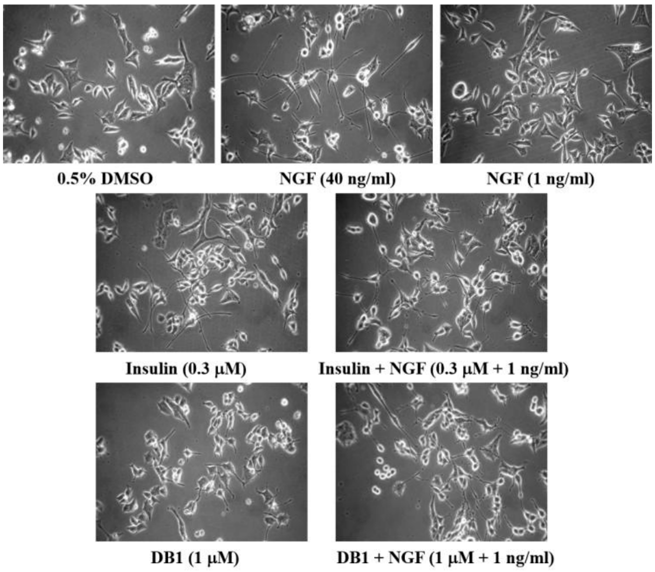

Supplementary Figure 7. Microphotograph of PC12 cells after treatment with insulin and Demethylasterriquinone B1.

Supplementary Table 1. List of inhibitors used in this study.

Protein	Inhibitor	Product No.	Tested concentration
TrkA	k252a	#82497, Sigma-Aldrich Co.	0.1 μM
TrkB	ANA-12	#S7745, Selleck	1 μM
INSR	HNMPA-[AM]$_3$	#sc-221730, Santa Cruz Biotechnology	1 μM
GR	RU486	#M8046, Sigma-Aldrich Co.	20 μM
PI3K	LY294002	#440202, Sigma-Aldrich Co.	10 μM
MEK/ERK	U0126	#19-147, Sigma-Aldrich Co.	20 μM
PLC	U73343	#S8011, Selleck	10 μM
PKC	Go6983	#G1918, Sigma-Aldrich Co.	1 μM
Ras	farnesylthiosalicylic acid	#10010501, Cayman Chemical	3 μM
Raf	AZ628	#sc-364418, Santa Cruz Biotechnology	3 μM

Supplementary Table 2. List of antibodies used in this study.

Primary antibody	Product No.	Tested dilution of primary antibody	Secondary antibody	Tested dilution of secondary antibody
Insulin receptor antibody	#3025, Cell Signaling Technology	1:1000	Goat Anti-Rabbit IgG, HRP Conjugated	1:5000
Phospho-insulin receptor antibody	#3024, Cell Signaling Technology	1:1000	Goat Anti-Rabbit IgG, HRP Conjugated	1:5000
AKT antibody	#9272, Cell Signaling Technology	1:1000	Goat Anti-Rabbit IgG, HRP Conjugated	1:5000
Phospho-AKT antibody	#4060, Cell Signaling Technology	1:1000	Goat Anti-Rabbit IgG, HRP Conjugated	1:5000
ERK1/2 antibody	#9102, Cell Signaling Technology	1:1000	Goat Anti-Rabbit IgG, HRP Conjugated	1:5000
Phospho-ERK1/2 antibody	#9101, Cell Signaling Technology	1:1000	Goat Anti-Rabbit IgG, HRP Conjugated	1:5000
PLC antibody	#2822, Cell Signaling Technology	1:1000	Goat Anti-Rabbit IgG, HRP Conjugated	1:5000
Phospho-PLC γ antibody	#2821, Cell Signaling Technology	1:1000	Goat Anti-Rabbit IgG, HRP Conjugated	1:5000
PKC antibody	#ab181558, Abcam	1:1000	Goat Anti-Rabbit IgG, HRP Conjugated	1:5000
Phospho-PKC antibody	#9371, Cell Signaling Technology	1:1000	Goat Anti-Rabbit IgG, HRP Conjugated	1:5000
GR antibody	#sc-393232, Santa Cruz	1:1000	Goat Anti-Mouse IgG, HRP Conjugated	1:5000
Phospho-GR antibody	#AF2004, Affinity BioReagents	1:1000	Goat Anti-Rabbit IgG, HRP Conjugated	1:5000
GAPDH Mouse Monoclonal Antibody	#CW0100, Cowin Biotech Company	1:1000	Goat Anti-Mouse IgG, HRP Conjugated	1:5000
